# Hypoxia Imaging and Biological Evaluation of the Radiosensitizing Effect of Oleanolic Acid

**DOI:** 10.1155/2018/2694679

**Published:** 2018-08-28

**Authors:** Hui Wang, Yu Zhang, Wenjing Yu, Yangyang Xue, Liang Xiao, Huiqin Xu

**Affiliations:** ^1^Department of Nuclear Medicine, The First Affiliated Hospital of Anhui Medical University, Hefei 230022, China; ^2^Department of Nuclear Medicine, Sir Run Run Shaw Hospital, Zhejiang University, Hangzhou 310016, China; ^3^Department of Radiotherapy, The First Affiliated Hospital of Anhui Medical University, Hefei 230022, China

## Abstract

**Background and Purpose:**

The aim of this study was to evaluate the radiosensitizing effect of oleanolic acid (OA) on C6 rat glioma and the changes in tumor biology during radiosensitization therapy on ^18^F-fluoromisonidazole (^18^F-FMISO) positron emission tomography/computed tomography (PET/CT).

**Methods:**

The radiosensitizing effect of OA on C6 tumors was assessed* in vivo* by measuring the tumor inhibitory rate and rat survival time. Meanwhile, rats with C6 tumors were imaged with ^18^F-FMISO PET/CT during radiosensitization therapy. Tumor-to-muscle ratio (TMR) of ^18^F-FMISO maximum uptake was calculated by region of interest analysis. Changes in tumor biology after therapy were assessed with immunohistochemical staining. ^18^F-FMISO uptake was analyzed in relation to expression of tumor biomarkers including hypoxia-inducible factor (HIF)-1*α*, glucose transporter (Glut-1), the proliferation antigen Ki67, tumor suppressor P53, and microvessel density (MVD).

**Results:**

The results showed that OA combined with radiation inhibited the growth rates of tumors and prolonged the survival period of tumor-bearing rats effectively (*χ*^2^ = 12.5,* p* < 0.01). ^18^F-FMISO PET/CT indicated decreases in hypoxia after radiosensitization therapy. Statistical differences were observed in TMR of the irradiation group and OA combined with irradiation group (*t* = 3.32,* p* < 0.05). HIF-1*α*, Glut-1, Ki67, P53, and MVD expressions in tumors were downregulated by OA combined with radiation as well as with radiation alone. Additionally, there was a significant positive linear correlation between TMR and HIF-1*α*, Glut-1, Ki67, P53, and MVD.

**Conclusions:**

These results suggest that OA has a radiosensitizing effect on C6 tumors in terms of tumor volume inhibition, survival extension, and multiple poor prognosis biological markers downregulation. ^18^F-FMISO PET/CT can be of value for tumor biology noninvasive capture and radiosensitization response evaluation.

## 1. Introduction

The 2015 Nobel Prize in Physiology or Medicine was awarded to Chinese scientist Professor Youyou Tu for her outstanding contribution to the discovery of the antimalarial effects of the Chinese herb* Artemisia annua L.* [1]. It is widely used, especially in Africa, reducing symptoms and saving the lives of malaria patients. Chinese herbal medicine and its natural derivatives have attracted the attention of researchers worldwide.

Traditional Chinese herbal medicine is one of the oldest medical systems on earth, with a history of more than 4,000 years. Oleanolic acid (3b-hydroxy-olea-12-en-28-oic acid, OA) has been isolated from several plants, including* Olea europaea L.*,* Glycyrrhiza sp.*,* Forsythia suspensa*,* Dendrobium sp*., and other plants [2, 3]. A pentacyclic triterpenoid compound, OA has been used in hepatitis and cirrhosis patients for over 20 years in China because of its hepatoprotective effect [4]. The pharmacological activity of OA, including antioxidant, antiinflammatory, hypoglycemic, and antineoplastic properties, is under investigation [5]. Recent studies have shown that OA significantly enhances the radiosensitivity of rat glioma C6 cells and human lung cancer A549 cells under hypoxic environment* in vitro* through the downregulation of intracellular glutathione (GSH) content and hypoxia-inducible factor-1*α* (HIF-1*α*) expression [6]. We have previously reported on the radiosensitizing effects of OA on C6 tumor-bearing rats monitored by ^18^F-FDG PET/CT [7]. Our aim in this study was to further assess the tumor hypoxia status and biological changes during OA radiosensitization therapy via ^18^F-FMISO PET/CT and immunohistochemical studies.

## 2. Materials and Methods

### 2.1. Cell Culture

C6 rat glioma cell line was cultured in Dulbecco's modified Eagle's medium (DMEM; GIBCO, USA) supplemented with 10% fetal bovine serum (FBS; HyClone, USA), 100 U/mL penicillin, and 100 *μ*g/mL streptomycin. C6 tumor cells were maintained at 37°C in a humidified 5% CO_2_ and 95% air.

### 2.2. Tumor Xenograft Rat Model Preparation

Four- to six-week-old male Sprague-Dawley (SD) rats, weighting 200~250 g, were provided by Anhui Medical University Animal Center. All rats were housed in a standardized environment at a temperature (20-25°C) and humidity (50 ± 5%) with a 12 h light/dark cycle and had access to food and water* ad libitum.*

All the experiment animal protocols in this study were approved by the Ethics Review Committee for Animal Experimentation of Anhui Medical University. SD rats were anesthetized by an intraperitoneal injection of chloral hydrate (mass fraction 10%, 0.3 mL/100 g); then C6 rat glioma cells (1.5 × 10^7^/mL, 200 uL tumor cells for each rat) were implanted subcutaneously in the right thigh region of SD rats. The experiment was initiated when the tumor length reached 1 cm.

### 2.3. Treatment Protocol

C6 tumor-bearing rats were randomized into four groups: (i) control group, treated with saline only; (ii) OA group, OA (purity > 99%, National Institute for the Control and Biological products, Beijing, CHINA) was prepared by suspension in DMSO solution (0.1% DMSO and 1.8% NaCl in distilled water), with a final concentration of 20 mg/mL. Tumor-bearing rats were given OA solution by gavage (40 mg daily for 7 days); (iii) irradiation (IR) group, tumor-bearing rats were irradiated once every two days over six days with a fractional dose of 2 Gy using a VARIAN 23 EX medical linear accelerator (Varian Medical System Inc., Palo Alto CA, USA); and (iv) OA combined with IR group, tumor-bearing rats were administered both OA and irradiation as described above.

### 2.4. Assessment of Tumor Volume and Survival Time

Six tumor-bearing rats in each of the four groups were observed for tumor growth and survival. Tumor size was measured by vernier caliper weekly. Tumor volume (V) was calculated as follows: V (cm^3^) = length (cm) × width^2^ (cm^2^) × 0.5. Tumor inhibitory rate was determined by the following formula: tumor inhibitory rate = (mean tumor volume of control group − mean tumor volume of treatment group)/mean tumor volume of control group. The survival of each rat was recorded and analyzed via the Kaplan-Meier method.

### 2.5. ^18^F-FMISO PET/CT Imaging

**Figure 1:**
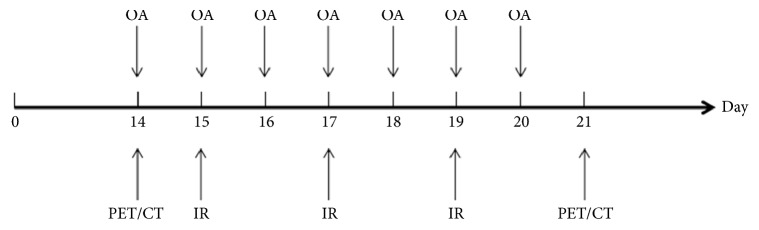
Outline of tumor implantation, treatment, and imaging schedule (OA: oleanolic acid, IR: irradiation at dose of 2 Gy, PET/CT: ^18^F-FMISO PET/CT, DAY: 0 is the tumor cells implantation day).

An additional six tumor-bearing rats in each group were investigated with ^18^F-fluoromisonidazole, radiochemical purity > 95% (^18^F-FMISO) PET/CT (Nanjing Jiangyuan Andike Positron Research and Development Co., Ltd., Wuxi, Jiangsu, China) imaging before and 24 h after treatment (Figure 1). All tumor-bearing rats were fasted for at least 4 hours. Each of the rats received 37 Mbq ^18^F-FMISO via tail vein injection. PET images were acquired 100–120 min after ^18^F-FMISO administration using PET/CT (Siemens Biograph 64 Truepoint PET/CT; Siemens, Germany). The CT parameters were as follows: 120 kV, 80 mA, and section thickness of 1.5 mm. PET acquisition was performed at 5 min per bed position.

### 2.6. PET/CT Image Data Analysis

The image plane with the largest tumor appearance on PET/CT fusion images was selected for data collection. Irregular regions of interest (ROIs) covering the entire tumor and the contralateral thigh muscle were drawn. The maximum standardized uptake value (SUVmax) of tumor and normal muscle were obtained automatically by the system. Tumor SUVmax/muscle SUVmax (TMR) was used as the PET/CT metric for statistical analysis.

### 2.7. Pathological Experiments

Tumor samples were dissected, formalin fixed, paraffin-embedded, and sectioned at a thickness of 4 *μ*m for hematoxylin and eosin staining and immunohistochemical staining. The expression level of tumor biological markers, including hypoxia-inducible factor- (HIF-) 1*α*, glucose transporter (Glut-1), the proliferation antigen Ki67, tumor suppressor P53, and microvessel density (MVD), were assessed by immunohistochemical studies using mouse polyclonal antibodies (HIF-1*α*: dilution 1:400, Santa Cruz, USA; Glut-1: dilution 1:300, Santa Cruz, USA; Ki67: dilution 1:500, Santa Cruz, USA; P53: dilution 1:200, Zhongshan Jinqiao, Beijing, China; MVD: polyclonal F antibody at dilution 1:400, Santa Cruz, USA). Immunoreactivities of HIF-1*α*, Glut-1, Ki67, and P53 were estimated by counting the percentage of positive cells per 100 tumor cells in the region with the greatest density of staining. Microvessel counting was performed in areas with maximal neovascularization within the tumors under 200× magnification. All the counts were made over five fields in each slide and the mean results were defined as the final value.

### 2.8. Statistical Analysis

All data are presented as mean ± standard derivation (SD). Statistical analysis was performed using commercial software (SPSS Version 17.0, Chicago, USA). The comparison among multiple groups was analyzed by one-way ANOVA and the comparison between two groups was analyzed by Student's* t*-test. Survival analysis was performed by the Kaplan-Meier method. The association between TMR and tumor markers was carried out with the Pearson correlation coefficient and linear regression.* p* < 0.05 was considered statistically significant.

## 3. Results

### 3.1. Radiosensitization Efficacy of OA in the C6 Rat Glioma Model

The radiosensitizing effect of OA on C6 tumor models was observed* in vivo* based on tumor volume inhibitory rates and survival time of tumor-bearing rats.

Before and 24 h after therapy, tumor volumes in the control group were (0.62 ± 0.05, 1.65 ± 0.09) cm^3^, tumor volumes in the OA group were (0.65 ± 0.04, 1.69 ± 0.11) cm^3^, tumor volumes in the IR group were (0.62 ± 0.04, 0.78 ± 0.19) cm^3^, and tumor volumes in OA combined with IR group were (0.63 ± 0.08, 0.81 ± 0.26) cm^3^ (Table 1). There was no significant difference between the control group and the OA group after treatment. Tumor inhibitory rates of the IR group and OA combined with IR group were 52.73% and 51.91%, respectively. There was no significant difference of tumor volume inhibitory effect between the IR group and the OA combined with IR group.

**Figure 2:**
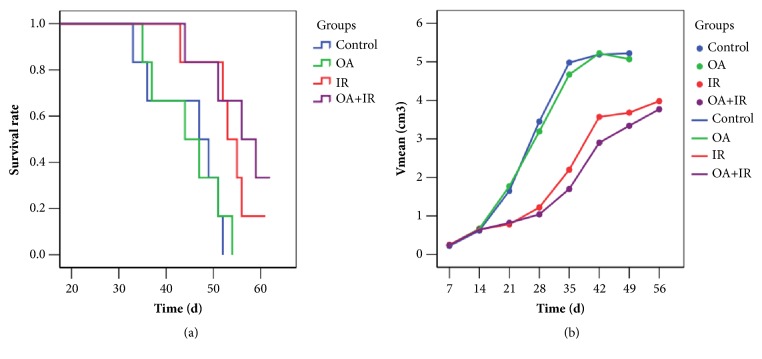
Enhanced antitumor effects of irradiation combined with OA. (a) Kaplan-Meier analysis of the survival of the tumor-bearing rats in the four groups. Sixty days after tumor cells inoculated into rats, survival rates of tumor-bearing rats in the control group, OA group, IR group, and OA combined with IR group were 0, 0, 16.7%, and 33.3%, respectively. (b) Tumor growth curve. Tumor volumes were inhibited in the IR group and OA combined with IR group; however, there was no significant difference between them.

The survival times of tumor-bearing rats in the control group, OA group, IR group, and OA combined with IR group were 43.67 ± 3.3 (range, 33–52) d, 44.62 ± 3.1 (range, 35–54) d, 53.3 ± 2.2 (range, 43–61) d, and 56.8 ± 2.6 (range, 44–65) d, respectively (Figure 2). Sixty days after tumor cells were inoculated into the rats, the survival rates of tumor-bearing rats in the control group, OA group, IR group, and OA combined with IR group were 0, 0, 16.7%, and 33.3%, respectively. The survival time differences among groups were statistically significant via Kaplan-Meier analysis (*χ*^2^ = 12.5, *p* < 0.01).

These results suggest that although there was no significant difference in tumor volume inhibitory rates between the IR group and the OA combined with IR group, OA enhanced the antitumor efficacy of radiation in form of prolonging the survival time.

### 3.2. ^18^F-FMISO PET/CT Imaging

**Figure 3:**
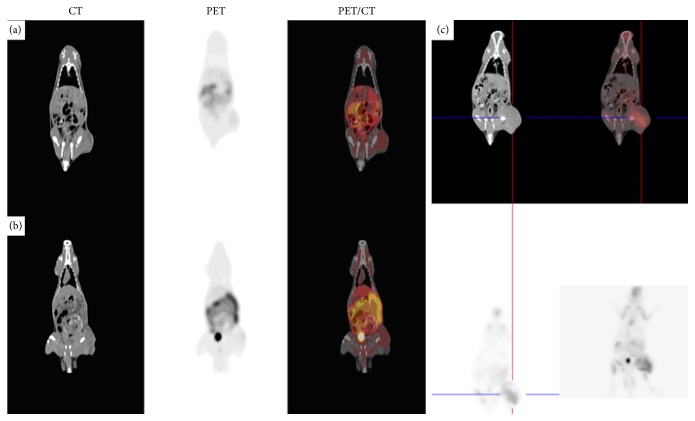
^18^F-FMISO PET/CT images. ((a) and (b)) From the OA combined with IR group, (a) before treatment, (b) 24 h after treatment, and (c) from the control group 24 h after treatment (cross wire indicates the tumor). TMR of the OA combined with IR group decreased slightly while increasing in the control group.

^18^F-FMISO PET/CT imaging was performed on each tumor-bearing rat before and 24 h after treatment (Figure 3). In this study, TMR of C6 tumor-bearing rats before therapy was (1.29 ± 0.12). The threshold for hypoxia based on TMR was set at 1.2; hypoxia was found in 91.7% (22/24) of C6 tumors imaged prior to therapy. 24 h after different therapies, the proportions of hypoxic tumors in the control group, OA group, IR group, and OA combined with IR group were 100% (6/6), 100% (6/6), 66.7% (4/6), and 33.3% (2/6), respectively.

**Figure 4:**
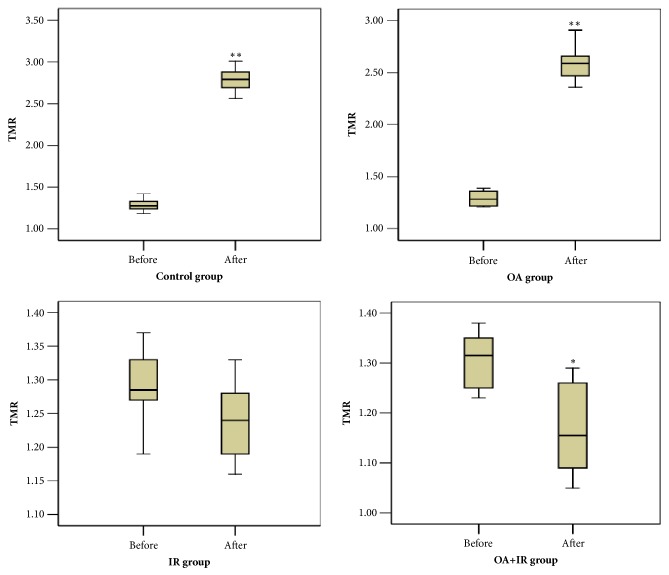
Changes in TMRs for the four groups after treatment (^*∗*^*p* < 0.05, ^*∗∗*^*p* < 0.01). The TMR of the control group and the OA group had increased after therapy. The TMR of the IR group and the OA combined with IR group had decreased slightly.

TMRs of each group before and 24 h after treatment were compared statistically. TMRs of the control group before and after treatment were (1.28 ± 0.08, 2.78 ± 0.16,* t* = 22.89,* p* < 0.01). TMRs of the OA group before and after treatment were (1.29 ± 0.16, 2.61 ± 0.22,* t* = 17.7,* p* < 0.01). TMRs of the IR group before and after treatment were (1.28 ± 0.06, 1.24 ± 0.32,* t* = 1.09,* p* > 0.05). TMRs of the OA combined with IR group before and after treatment were (1.30 ± 0.07, 1.15 ± 0.13,* t* = 3.26,* p* < 0.05) (Table 2 and Figure 4). TMR growth rates of the control and OA groups were 117.18% and 102.32%, respectively. TMR rates of the IR group and the OA combined with IR group were 3.12% and 11.54%, respectively.

### 3.3. Pathological Experiments

Gross observation showed that the C6 tumors were round and nodular with clear boundaries. After incision, the margin of tumor was like reddish fish and the central region was like grayish white surimi.

H&E staining showed obvious heterogeneity, large nuclei, and dense and disordered arrangement of cells. The necrotic area was red stained. Irreversible damage to tumor cells was found in the IR group and the OA combined with IR group in the form of nuclear pyknosis, nucleolysis, nuclear rupture, and so on.

**Figure 5:**
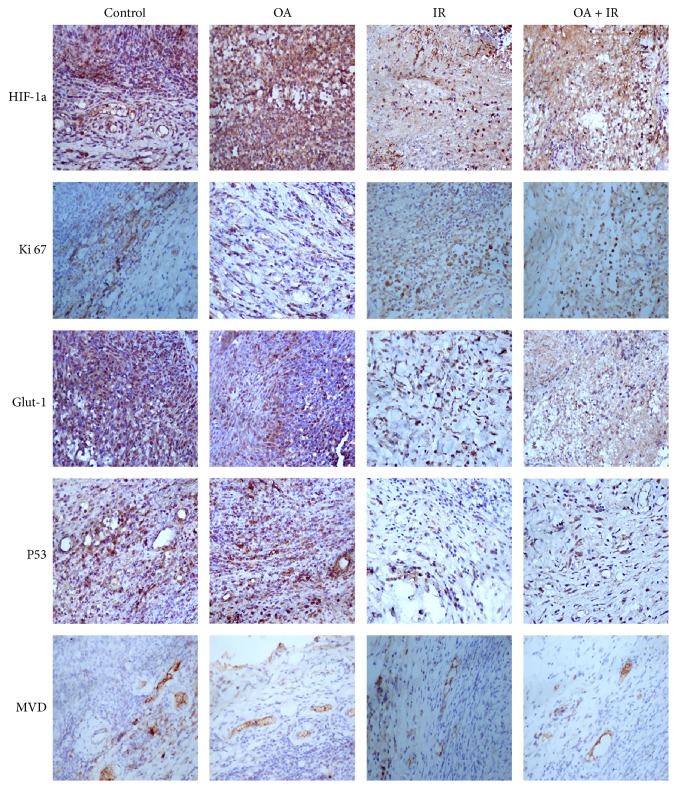
Immunohistochemical staining of multiple tumor biological markers after treatment. HIF-1*α*, Glut-1, Ki67, P53, and MVD expressions in tumors were downregulated in the IR group and the OA combined with IR group. HIF-1*α* = hypoxia-inducible factor, Glut-1 = glucose transporter, Ki67 = proliferation antigen, P53 = tumor suppressor, and MVD = microvessel density.

Immunohistochemical analysis was carried out to assess tumor biological indicators including HIF-1*α*, Glut-1, Ki67, P53, and MVD (Figure 5). The percentages of HIF-1*α* positively stained cells in the control group, OA group, IR group, and OA combined with IR group were 84.53 ± 4.91, 81.82 ± 6.45, 52.53 ± 6.87, and 38.87 ± 6.32%, respectively. The percentages of Glut-1 positive cells in the control group, OA group, IR group, and OA combined with IR group were 89.24 ± 5.88, 89.87 ± 3.94, 67.25 ± 9.76, and 45.09 ± 9.61%, respectively. The percentages of Ki67 positive cells in the control group, OA group, IR group, and OA combined with IR group were 35.57 ± 4.39, 36.52 ± 4.62, 19.53 ± 6.78, and 15.85 ± 4.83%, respectively. The percentages of P53 positive cells in the control group, OA group, IR group, and OA combined with IR group were 53.06 ± 10.41, 52.84 ± 11.93, 27.79 ± 12.45, 30.38 ± 13.02%, respectively. MVD of tumors in the control group, OA group, IR group, and OA combined with IR group were 19.01 ± 5.34, 18.37 ± 4.98, 9.06 ± 3.35, and 9.67 ± 3.68, respectively. Significant differences in these tumor biological indicators were observed between groups (*F* = 78.83, 45.94, 25.43, 7.98, 9.25,* p* < 0.01, respectively). Pairwise comparison was performed via least significant difference (*LSD*) statistical analysis. There were no significant differences in HIF-1*α*, Glut-1, Ki67, P53, or MVD between the control group and the OA group. The percentages of HIF-1*α* and Glut-1 positive cells in the OA combined with IR group were lower than in the IR group (*p* < 0.05). However, there was no significant difference in Ki67, P53, or MVD between the OA combined with IR group and the IR group.

### 3.4. The Association between ^18^F-FMISO Uptake and Tumor Markers

As an index for ^18^F-FMISO uptake, TMR showed positive correlation with tumor biological indicators including HIF-1*α*, Glut-1, Ki67, P53, and MVD (*r* = 0.92, 0.86, 0.89, 0.70, 0.81,* p* < 0.01) by Pearson correlation coefficient analysis. TMR was used as a dependent variable, and HIF-1*α*, Glut-1, Ki67, P53 and MVD were used simultaneously as independent variables for multivariate linear regression analysis. The results indicated that the percentage of HIF-1*α* positive cells in tumor tissue was an independent factor for TMR value (*p* < 0.01) (Table 3).

## 4. Discussion

Radiotherapy is one of the primary treatments for cancers. Tumor hypoxia has been identified as a major negative independent prognostic factor influencing tumor response to radiotherapy [8]. Hypoxic tumor cells are associated strongly with tumor resistance to radiation and subsequent development of recurrence and metastases. Recently, OA has been proven to have a radiosensitizing effect on hypoxic tumor cells through the downregulation of intracellular GSH content and HIF-1*α* expression [6, 9]. The radiosensitizing effect of OA monitored by ^18^F-FDG PET/CT has been previously evaluated by our lab. We demonstrated that the addition of OA to radiation significantly inhibited the glucose uptake and tumor growth. Considering the radiosensitization mechanism of OA related to hypoxia, ^18^F-FMISO PET/CT, as a noninvasive and sensitive method to detect tumor hypoxia, would provide more information about tumor vulnerability during radiosensitization therapy.

In the present study, to our knowledge, we reported for the first time the radiosensitization effect of OA via ^18^F-FMISO PET/CT imaging. Our results showed that OA combined with radiation could inhibit tumor growth and prolong the survival of tumor-bearing rats. However, tumor volume did not shrink immediately after therapy and there was no significant difference compared with the IR group. Two aspects need to be considered. First, the purpose of our research was to evaluate the early radiosensitizing effect of OA; both the dose and duration of OA and radiation therapy were insufficient to destroy a tumor. Second, tumor volume is not a sensitive indicator for early efficacy evaluation because of the various influential factors like the clearance rate of apoptotic tumor cells, the infiltration of inflammatory cells, the component of fibrosis, etc. [10]. OA enhanced antitumor efficacy of radiation in form of prolonging the survival time of rats.

Nuclear medicine imaging techniques, especially PET/CT, have long been recognized in clinical practice for cancer diagnosis and efficacy evaluation. ^18^F-FMISO is a nitroimidazole compound radiolabeled with ^18^F which should accumulate in hypoxic tissue and wash out quickly from normoxic tissue [11]. ^18^F-FMISO has been widely used as a PET imaging tracer of hypoxia [12]. Compared with ^18^F-FDG, the signal of ^18^F-FMISO in tumors is lower. The SUVmax in tumor and the tumor-to-muscle or tumor-to-blood SUVmax ratio (TMR or TBR) are common hypoxia metrics in ^18^F-FMISO PET/CT imaging. Much clinical and preclinical evidence suggest that the threshold for hypoxia based on TMR or TBR should be set at 1.2–1.6 depending on the type of tumor. Tumor areas with TMR or TBR > 1.2–1.6 have been confirmed to be hypoxic by oxygen electrode measurement or pimonidazole immunohistochemistry staining [13–17].

In this study, with the threshold for hypoxia based on TMR set at 1.2, hypoxia was found in 91.7% (22/24) C6 tumors imaged prior to therapy. This agrees with other reports that hypoxia is prevalent in solid tumors [18]. 24 h after therapy, the proportions of hypoxic tumors in the IR group and OA combined with IR group were significantly lower than in the control group and OA group. TMR decreased in the IR group and OA combined with IR group after therapy. By contrast, TMR increased in the control group and OA group. That the decline of TMR in the OA combined with IR group was greater than that in the IR group confirms that OA combined with radiation in more effective than radiation alone, although OA is not a good agent for chemotherapy. Irreversible damage of tumor cells such as nuclear pyknosis, nucleolysis, and nuclear rupture was found in the IR group and the OA combined with IR group. The ^18^F-FMISO PET/CT imaging results and HE staining confirmed our understanding of the efficacy of radiosensitization therapy.

The hypoxic microenvironment of tumor cells is the most important factor in radiotherapy resistance. As a key transcriptional factor activated by hypoxia, HIF-1*α* induces the expression of many transcription factors including Glut-1, Ki67, P53, and angiogenesis-related genes. These regulate glucose metabolism, cell survival, apoptosis, angiogenesis, and so on [19–24]. Many recent studies have provided evidence of strong correlation between HIF-1*α* and its target genes with tumor resistance, metastasis, angiogenesis, poor prognosis, etc. [25–28]. Monitoring the biological behavior of tumors may benefit patients by allowing for more personalized treatment strategy. In the present study, multiple poor prognosis biological markers including HIF-1*α*, Glut-1, Ki67, P53, and microvessel density (MVD) in tumors were downregulated in unison after radiosensitization therapy. The percentage of HIF-1*α* and Glut-1 positive cells in the OA combined with IR group was statistically significantly lower than in the IR group, suggesting that radioresistance in the OA combined with IR group was lowest owing to the radiosensitization effect of OA. The primary mechanism of this radiosensitizing effect involves the downregulation of HIF-1*α* and its target genes like Glut-1, Ki67, P53, angiogenesis-related genes, and so on, regulation of hypoxia, glucose metabolism, proliferation, metastasis, and neovascularization in tumors.

TMR of ^18^F-FMISO showed positive linear correlation with HIF-1*α*, Glut-1, Ki67, P53, and MVD in varying degrees, suggesting that ^18^F-FMISO PET/CT can be of value for tumor biology capture. TMR was set as a dependent variable, and HIF-1*α*, Glut-1, Ki67, P53, and MVD were used simultaneously as independent variables for multivariate linear regression analysis. The results indicated that the percentage of HIF-1*α* positive cells in tumor tissue was an independent factor for TMR value. This confirms the hypoxia specificity of ^18^F-FMISO and suggests ^18^F-FMISO PET/CT as a potential noninvasive method to evaluate the hypoxic status of tumors.

## 5. Conclusion

The present experiments indicate that OA has radiosensitizing effect on C6 tumor in terms of tumor volume inhibition, survival extension, and downregulation of multiple poor prognosis biological markers when combined with radiotherapy. ^18^F-FMISO PET/CT can be of value for tumor biology noninvasive capture and radiosensitization response evaluation.

## Figures and Tables

**Table 1:** Tumor volumes and tumor inhibition rates before and after treatment (n = 6 per group).

Group	Tumor volume (cm^3^)		Tumor inhibition rate
	Before treatment	24 h after treatment	
Control	0.62 ± 0.05	1.65 ± 0.09^*∗∗*^	
OA	0.65 ± 0.04	1.69 ± 0.11^*∗∗*^	
IR	0.62 ± 0.04	0.78 ± 0.19	52.73%
OA+IR	0.63 ± 0.08	0.81 ± 0.26	51.91%

The growth rates in IR group and OA combined with IR group were restrained after therapy. Compared with IR group: ^*∗∗*^*p < 0.01, *^*∗*^*p < 0.05*.

**Table 2:** TMR of each group before and after treatment. (n = 6 per group).

Group	TMR		ΔTMR
	Before treatment	24 h after treatment	
Control	1.28 ± 0.08	2.78 ± 0.16^*∗∗*^	117.18%
OA	1.29 ± 0.16	2.61 ± 0.22^*∗∗*^	102.32%
IR	1.28 ± 0.06	1.24 ± 0.32	- 3.12%
OA+IR	1.30 ± 0.07	1.15 ± 0.13^*∗*^	- 11.54%

TMR was the lowest in the OA combined with IR group among the four groups after therapy. Compared with IR group: ^*∗∗*^*p < 0.01, *^*∗*^*p < 0.05*.

**Table 3:** Multivariate linear regression analysis of TMR and tumor biological indices.

Variable	Standardized coefficient	*t*	*P*
HIF-1a (%)	0.554	3.575	**0.002**
Glut-1 (%)	-0.059	- 0.302	0.766
Ki67 (%)	0.267	1.380	0.185
P53 (%)	0.053	0.528	0.604
MVD	0.218	2.004	0.060

The percentage of HIF-1*α* positive cells in tumor tissue was indicated as an independent factor for TMR value (*p* < 0.01).

## Data Availability

The data used to support the findings of this study are available from the corresponding author upon request.
